# PEGylated dendrimer-entrapped gold nanoparticles with low immunogenicity for targeted gene delivery†

**DOI:** 10.1039/c7ra11901a

**Published:** 2018-01-03

**Authors:** Bei Xu, Aijun Li, Xinxin Hao, Rui Guo, Xiangyang Shi, Xueyan Cao

**Affiliations:** College of Chemistry, Chemical Engineering and Biotechnology, Donghua University Shanghai 201620 People's Republic of China xshi@dhu.edu.cn caoxy_116@dhu.edu.cn

## Abstract

The high efficiency and specificity of gene therapy are mainly ascribed to the excellent transfection ability of the gene carrier. Non-viral polymer vectors have attracted extensive attention because of their low cytotoxicity and outstanding genetic loading capacity compared with viral vectors. For safe and efficient transfection of nuclear acids, here we report a novel gene delivery system, dendrimer-entrapped gold nanoparticles modified with a folate-conjugated poly (ethylene glycol) (Au DENPs-PEG-FA), possessing superior gene transfection efficiency than that of partially hydrophilic methoxy poly(ethylene glycol) (mPEG)-modified dendrimer-entrapped gold nanoparticles (Au DENPs-mPEG). The prepared Au DENPs-PEG-FA were well characterized, and our data revealed that the vector showed good cytocompatibility. Additionally, the quantification of inflammatory cytokines detected by qRT-PCR showed that the vectors displayed low innate immune response. The efficiency of nucleic acid (encoding enhanced green fluorescent protein (EGFP) and luciferase (Luc) reporter) transfection evaluated *via* flow cytometry and confocal microscopic imaging suggested that the Au DENPs-PEG-FA were able to transfect nucleic acid into HeLa cells with enhanced transfection efficiency. Furthermore, the existence of FA rendered the Au DENPs with excellent targeting performance *via* FA receptor-ligand binding interaction. The designed Au DENPs-PEG-FA with low immunogenicity and enhanced gene transfection efficiency may hold a great promise to be a superior vector for gene therapy.

## Abstract 2

The designed Au DENPs-PEG-FA compacts pDNA into cells to enhance gene transfection efficiency.

## Graphical abstract

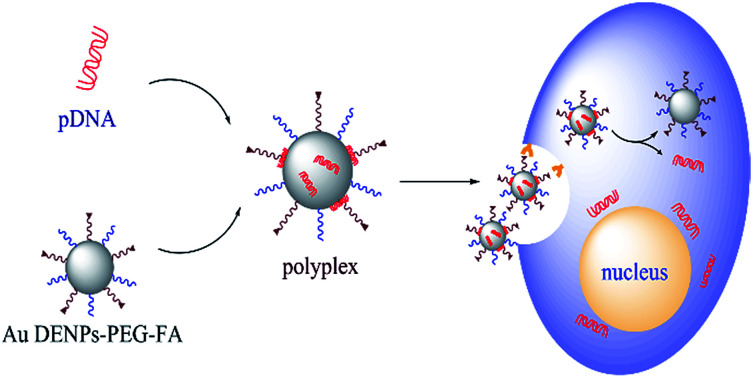

## 1 Introduction

Cancer represents a disease with high incidence and is therefore the main research object for gene therapy. Gene therapy for cancer can ideally kill tumor cells or inhibit its growth selectively by using nucleic acids as the therapeutic agent. Currently most of therapeutic genes are transfected into the tumor cells by viral or nonviral vector systems.^1^ The capacity of virus vectors to carry nucleic acid molecular is limited that mass production of them is difficult,^2^ limiting severely their applications *in vitro* and *in vivo*. By contrast, nonviral transfection is more advantageous and attractive. Synthetic carriers such as polycations have been proposed as efficient and safe alternative to viral systems for gene delivery.^3,4^ Due to the high cytotoxicity, the surface of polycations, such as polyamidoamine (PAMAM) dendrimers, need to be modified. The presence of large amounts of amine groups on the surface of dendrimers allow them to be surface modified through PEGylation,^5^ acetylation^6^ or alkylation,^7^ in which PEGylation has been most widely used. Jevprasesphant *et al.*^8^ and Luo *et al.*^9^ have proved that modification of PEG can reduce the cytotoxicity of PAMAM vector and significantly improve the efficiency of gene transfection.

Apart from cytotoxicity, another side effect of unwanted activation of the immune system also limit the further applications of nanomaterials in gene therapy. Unwanted activation of innate immune response can be a obstacle for biopharmaceutical development, as patients may develop local swelling and symptoms resembling influenza infection (*e.g.*, headache, fever, myalgia, and nausea).^10^ Much effort has been made on evaluating the risk for immunological safety of nanoparticles and macromolecular biomaterials in living organisms.^11,12^ Nevertheless, the immunogenicity of dendrimers packaged with nanoparticles after transfection of nucleic acid has not been investigated. Zhang *et al.*^13^ suggested that nanoparticles could induce the production of free radicals and then affect biotoxicity. Some materials at present, such as carbon nanomaterials^14,15^ and so on, was found to be highly immunogenic and can cause inflammation. Therefore, the immunogenicity should be considered as a factor in clinical research.

By using the high affinity of folic acid (FA) molecules to the surface folate receptors on tumor cells, such as HeLa cells, KB cells and so on, FA-conjugated compounds can deliver polyplexes with different sizes to pathological cells without damaging normal tissue. The dendrimers modified with FA can improve the effectiveness of gene delivery.^16^ The PAMAM packing gold nanoparticles can support the relative 3D morphology of PAMAM dendrimers,^17^ contributing to enhanced compaction of the nucleic acid and to easy transfection into cells.^16^

**Scheme 1:**
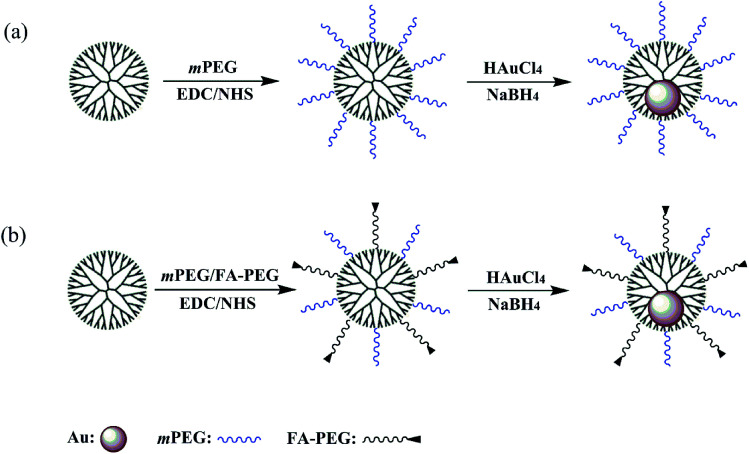
Synthesis of {(Au^0^)_50_-G5.NH_2_-mPEG_10_} (a) and {(Au^0^)_50_-G5.NH_2_-PEG_10_-FA_5_} (b).

In this work, we explored a new targeted gene delivery system with low cytotoxicity and immunogenicity. Amine-terminated generation 5 (G5) dendrimers (G5.NH_2_) were modified with the carboxyl groups of mPEG and FA (Scheme 1b). The mPEG-modified G5.NH_2_ were also synthesized (Scheme 1a) for comparison. The polyplexes were synthesized by mixing the vectors with nucleic acid at three different N/P ratios, namely 1 : 1, 2.5 : 1 and 5 : 1, respectively. The surface potential and particle size of the polyplexes were measured by dynamic light scattering and zeta potential measurements. The encapsulation capacity of the nucleic acid was determined by agarose gel retardation assay. The cytotoxicity of the vectors was evaluated by cell counting kit-8 (CCK-8). And qRT-PCR was used to quantitatively measure the inflammatory cytokines. The functionalized dendrimers modified with FA are low immunogenic and can realize the targeted gene delivery to transfer both EGFP gene and Luc reporter gene to HeLa cells.

## 2 Experimental section

### 2.1 Materials

G5 PAMAM dendrimers were purchased from Dendritech, Inc. (Midland, USA). PEG with one end of amine group and the other end of carboxyl group (COOH-PEG-NH_2_, *M*_w_ = 2000), and PEG monomethyl ether with one end of carboxyl group (mPEG-COOH, *M*_w_ = 2000) were purchased from Shanghai Yanyi Biotechnology Corporation (Shanghai, China). FA, 1-(3-dimethylaminopropyl)-3-ethylcarbodiimide hydrochloride (EDC), dulbecco modified eagle medium (DMEM), fetal bovine serum (FBS), and cell counting kit 8 (CCK-8) were supplied by Sigma-Aldrich (St. Louis, MO). LB broth, agarose and EZ Spin Column Plasmid Mini-Preps Kit were from Sangon Biological Engineering Technology Co., Ltd (Shanghai, China). Dimethylsulfoxide (DMSO) was from Lingfeng Chemical Reagent Co., Ltd. (Shanghai, China). Primary Amino Nitrogen (PANOPA) assay kit was supplied by Megazyme International Ltd. (Wicklow, Ireland). Luciferase assay kit was purchased from Promega (Fitchburg, WI). The nucleic acid labeling kit containing Cy3 reagent was supplied by Mirus Bio LLC (Madison, WI). HeLa cells, L929 cells and macrophage cells (RAW 264.7) were obtained from the Shanghai Institutes for Biological Sciences, the Chinese Academy of Sciences. The pDNA encoding for Luc or EGFP were purchased from Promega (Fitchburg, WI). CpG DNA was acquired from Sangon Biological Engineering Technology Co., Ltd (Shanghai, China). Trizol and Premium Reverse Transcriptase were purchased from Thermo Scientific Inc. (Waltham, MA). SuperReal PreMix Plus was from Tiangen Biotech Co., Ltd. (Beijing, China).

### 2.2 Synthesis of Au DENPs-mPEG and Au DENPs-PEG-FA

mPEG-COOH (*M*_w_ = 2000), EDC, and *N*-hydroxysuccinimide were homogeneously mixed at a molar ratio of 1 : 10 : 10 and reacted for 30 min in dimethyl sulfoxide (DMSO, 5 mL). The activated mPEG was mixed with G5.NH_2_ in DMSO at a molar ratio of 10 : 1 and reacted for 72 h. HAuCl_4_ was added to the above product and mixed for 30 min to make the molar ratio of dendrimer to gold to be 1 : 50. Then NaBH_4_ was added to G5.NH_2_-mPEG_10_/HAuCl_4_ mixture solution and reacted for 3 h under stirring. The reaction solution was dialyzed against pure water with a MWCO of 10 000 for 3 days, and finally was freeze-dried to obtain {(Au^0^)_50_-G5.NH_2_-mPEG_10_} (P1) samples. The FA and EDC were homogeneously mixed at a molar ratio of 1 : 0.9, reacted for 30 min, and then reacted with NHS for 3 h. mPEG, G5.NH_2_ and the above activated FA were mixed at a molar ratio of 10 : 1 : 5 and reacted for 72 h to obtain {(Au^0^)_50_-G5.NH_2_-PEG_10_-FA_5_} (P2) sample.

### 2.3 Characterization of vectors

P1 and P2 were dissolved in D_2_O, and then characterized by ^1^H NMR. The formed materials were measured by ultraviolet-visible (UV/Vis) spectrophotometry. To further confirm the size of P1 and P2, TEM was performed with a JEOL 2010F analytical electron microscope (Tokyo, Japan) at 200 kV. The samples were prepared by dripping the aqueous solutions on the surface of the carbon-coated copper mesh, then dried at room temperature. The size distribution histogram was measured using Image J software.

### 2.4 Cytotoxicity of vectors

Cell counting kit-8 (CCK-8) assay was used to detect the cytotoxicity of P1 and P2 incubated with HeLa cells at different concentrations. HeLa cells were seeded in 96-well plates at a density of 6 × 10^3^ cells per well, and incubated in 200 μL DMEM containing 10% FBS, penicillin (100 units per mL), streptomycin (100 μg mL^−1^) at 37 °C and 5% CO_2_ for 24 h. The medium was changed to fresh serum-free medium containing various concentrations of vectors ranging from 50 nM to 3000 nM, and further incubated for another 24 h. Then 10 μL of CCK-8 (5.0 mg mL^−1^) was added to each well and incubated in the incubator for 4 h. Finally, the absorbance of each sample with 5 replicates at 450 nm was measured with a microplate reader (Thermo Fisher Scientific).

### 2.5 Immunogenicity of vectors and polyplexes

Real-time quantitative reverse transcription PCR (real-time qRT-PCR) assay was used to test the relative expression of inflammatory cytokines upon incubation with P1 and P2 in mRNA level. RAW 264.7 cells were plated at a density of 2 × 10^5^ cells per well in 24-well plate and cultured overnight. Cells were incubated with P1, P2 and lipofectamine in serum-free medium for 24 h. After incubation, the total RNA of each sample was extracted using TRIzol Reagent (Invitrogen) following the manufacturer's protocols. The cDNA products were synthesized with primers and Revert Aid Premium Reverse Transcriptase (Thermo Scientific) from the total RNA. The obtained cDNA was amplified with SuperReal PreMix Plus Kit on the quantitative real-time PCR detecting instrument (Bio-RAD) for detecting the cytokines of TNF-α and IL-6. The results were normalized to GAPDH expression. The relative expression level was calculated by 2^−ΔΔCT^ method, and the primer sequences were based on the reference sequences.^18^

### 2.6 Determination of the number of primary amines of vectors

The materials were dissolved in pure water. Then the number of the primary amines were examined by PANOPA assay according to the manufacturer's instructions.

### 2.7 Gel retardation assay

The pDNA binding ability of different vectors was determined by agarose gel retardation assay. The vectors (2 mg mL^−1^) and pDNA (549 ng μL^−1^) polyplexes were prepared under different N/P ratios with 1 μg pDNA, and incubated at room temperature for 30 min. The naked pDNA (1 μg) was used as a control. Agarose gel (1.0% w/v) was prepared in Tris–borate–EDTA buffer. The gel electrophoresis was performed at a voltage of 80 V for 30 min. The migration of pDNA in the gel was analyzed by a FR-1000 gel image analysis system (Shanghai Furi Science & Technology Co., Ltd.).

### 2.8 Dynamic light scattering (DLS) and zeta potential measurements

DLS measurements of vectors and vector/pDNA were performed on a Zetasizer Nano ZS system (Malvern, UK) instrument with a standard 633 nm laser. Each vectors/pDNA polyplex was prepared under the N/P ratios of 1 : 1, 2.5 : 1, and 5 : 1 with 1 μg pDNA.

### 2.9 Evaluation of green fluorescent protein (EGFP) expression

Fluorescence microscopic analysis was used to detect the gene transfection efficiency of the two vectors delivering EGFP genes using a Nikon Eclipse TE 2000E inverted microscope under three different N/P ratios. HeLa cells were seeded in 24-well plates at a density of 3 × 10^4^ cells. The cultured cells with vector/pDNA polyplexes at an N/P ratio of 1 : 1, 1 : 2.5 and 1 : 5 were transfected for 24 h, respectively. Finally, the cells were observed by fluorescence microscopy.

### 2.10 Evaluation of Luc gene transfection experiment

This experiment was also carried out to quantitatively examine the gene transfection efficacy. Similarly, the polyplexes were prepared by P1 or P2 and Luc pDNA, and incubated with HeLa cells for 24 h. Then, the cells were harvested and Luc activities were analyzed according to the protocol of Promega's Luc assay. The fluorescence value was tested by the automatic multimode reader (Bio-TEK). The gene transfection efficiency was determined in the fluorescence unit of each mg protein (RLU per mg). The non-transfected cells and the cells transfected with naked pDNA were used as negative control.

### 2.11 Cellular uptake of vector/pDNA polyplexes

Cy3-labeled pDNA was used to determine the intracellular uptake capacity of polyplexes. HeLa cells were planted with a density of 1 × 10^5^ cells on a 12-well plate and cultured overnight. Then the vector/pDNA polyplexes with 1 μg pDNA were added and the cells were incubated for 4 h. After that, the cells were washed three times with PBS. Glutaraldehyde (2.5% w/v) was added and the cells were fixed for 30 min at 4 °C. DAPI (1 μg mL^−1^) dye was used to stain the nucleus. The results were observed and recorded using a laser confocal microscope (LSM-700, Jena, Germany).

Flow cytometry was used to further detect the uptake capacity quantitatively. Similar to the above experiment, the treated cells were resuspended with 1 mL of sterilized PBS and analyzed by flow cytometry. The fluorescence of each cell sample was measured in the FL2-H channel. Three parallel experiments were performed in each group. The uptake efficiency of polyplexes in L929 was also detected according to the above methods.

### 2.12 The folate receptor (FAR) shielding experiment

The FAR shielding experiment was conducted to investigate the influence of the FAR-blocked cells on cell uptake efficiency. In this experiment, cells with 1 × 10^5^ per well were planted on 24 well plate and cultivated under the condition of 37 °C and 5% CO_2_ for 12 hours. In the preset three N/P conditions, mixture was prepared by mixing 1 μg pDNA, FA (10 μM) and the materials (2 mg mL^−1^) in each well for 20 min and was stored away from light at room temperature. After that, replace the medium with a fresh medium without FBS, then the cells were transfected in the plate for 4 hours. The cells were digested by trypsin and washed with 1 mL of sterile PBS and store the tested samples on ice. Each sample was tested by flow cytometry. Three parallel experiments were performed in each group.

### 2.13 Statistical analysis

One way ANOVA statistical analysis was performed to evaluate the significance of the experimental data. 0.05 was selected as the significance level, and the data were indicated with (*) for *p* < 0.05, (**) for *p* < 0.01, and (***) for *p* < 0.001, respectively.

## 3 Results and discussion

### 3.1 Synthesis and characterization of vectors

**Fig. 1:**
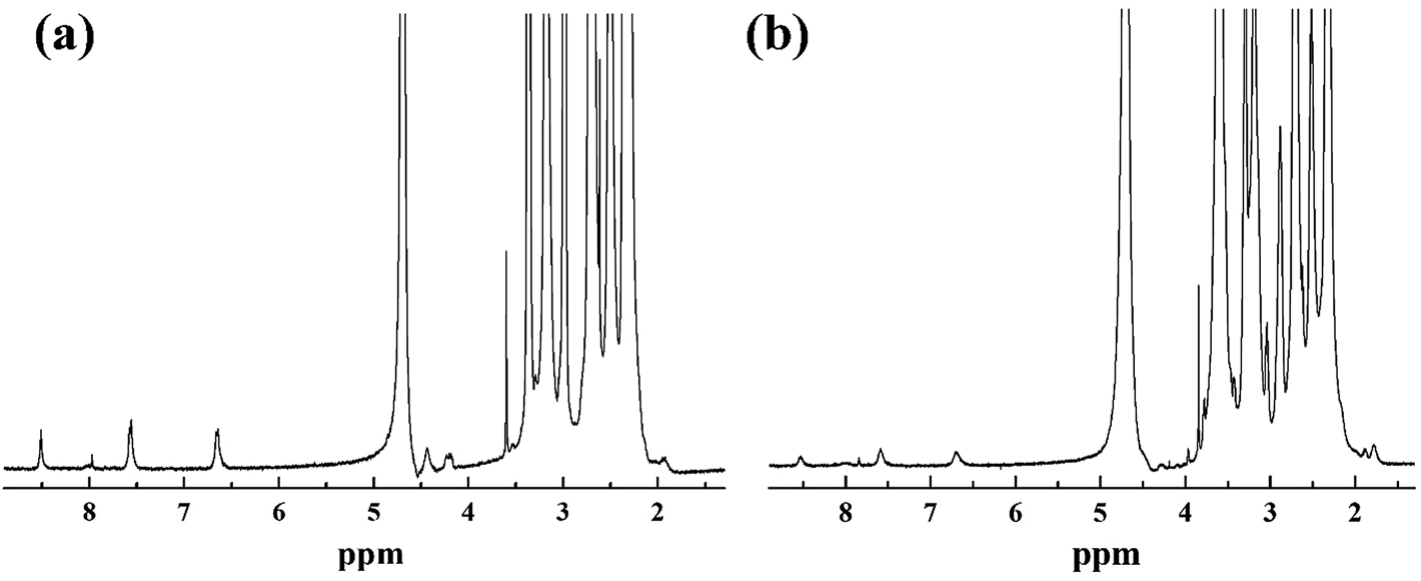
^1^H-NMR spectrum of {(Au^0^)_50_-G5.NH_2_-mPEG_10_} (a) and {(Au^0^)_50_-G5.NH_2_-PEG_10_-FA_5_} (b).

**Fig. 2:**
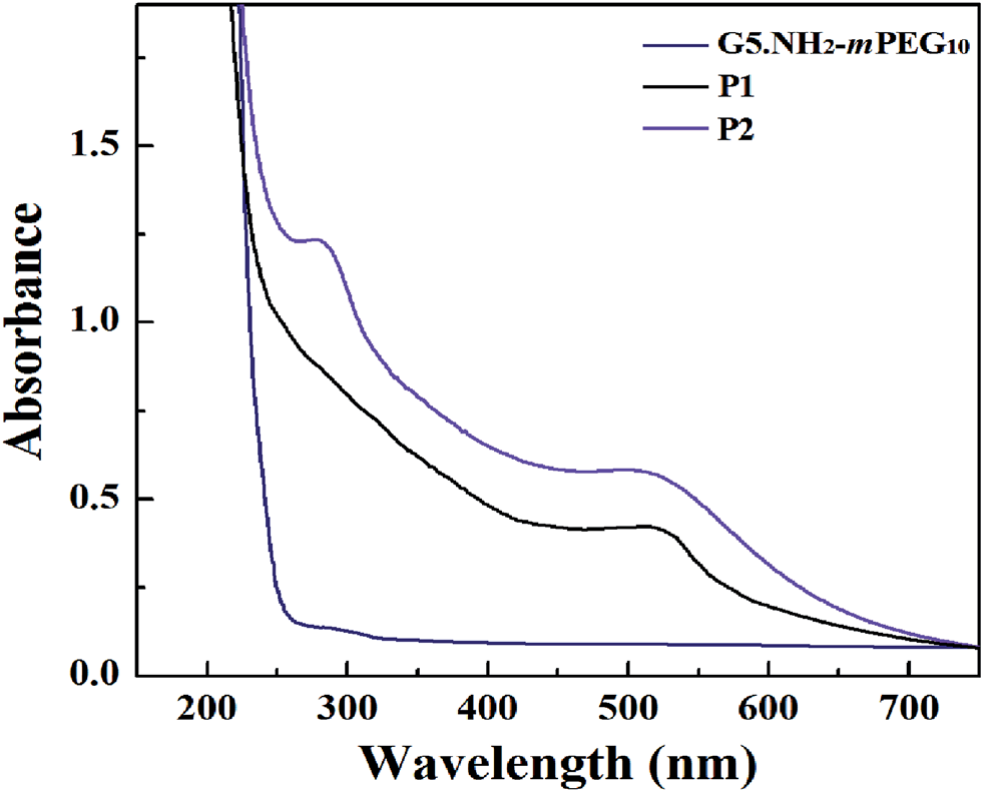
UV-Vis absorption spectra of {G5.NH_2_-mPEG_10_}, {(Au^0^)_50_-G5.NH_2_-mPEG_10_} (P1) and {(Au^0^)_50_-G5.NH_2_-PEG_10_-FA_5_} (P2).

**Fig. 3:**
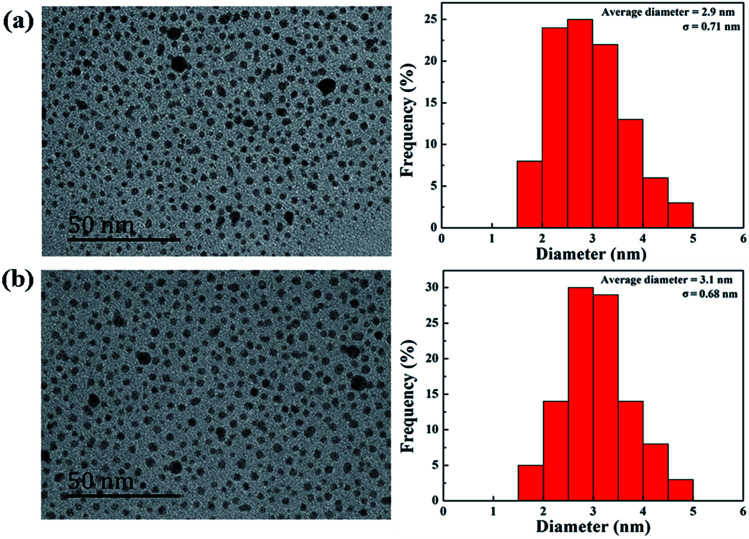
TEM image and average size distribution histogram of {(Au^0^)_50_-G5.NH_2_-mPEG_10_} (a) and {(Au^0^)_50_-G5.NH_2_-PEG_10_-FA_5_} (b).

According to our previous studies,^19,20^ we successfully prepared Au DENPs-PEG-FA by using G5.NH_2_ dendrimers as a template, and Au DENPs-mPEG were also synthesized as control (Scheme 1). As shown in Fig. 1, the proton peaks between 2.0 and 3.2 ppm showed that mPEG was successfully connected to dendrimer. The peaks between 6.6 and 8.5 indicated that FA was successfully grafted to dendrimer. By integrating the area, 9.8 mPEG (*M*_w_ = 2000) and 5 FA were calculated to be grafted onto each dendrimer, respectively. According to the UV/Vis spectrophotometry, the absorption peak at 520 nm for two materials can be assigned to the surface plasmon resonance (SPR) band of Au NPs, confirming the successful formation of Au DENPs (Fig. 2). The absorption peaks at 280 nm imply that the FA has been successfully modified. In addition, the two different vectors were characterized by TEM. The average Au core size of the P1 and P2 was estimated to be 2.9 nm and 3.1 nm, respectively (Fig. 3). They are spherical, small in size and have a narrow size distribution.

### 3.2 Cytotoxicity and immunogenicity of vectors

**Fig. 4:**
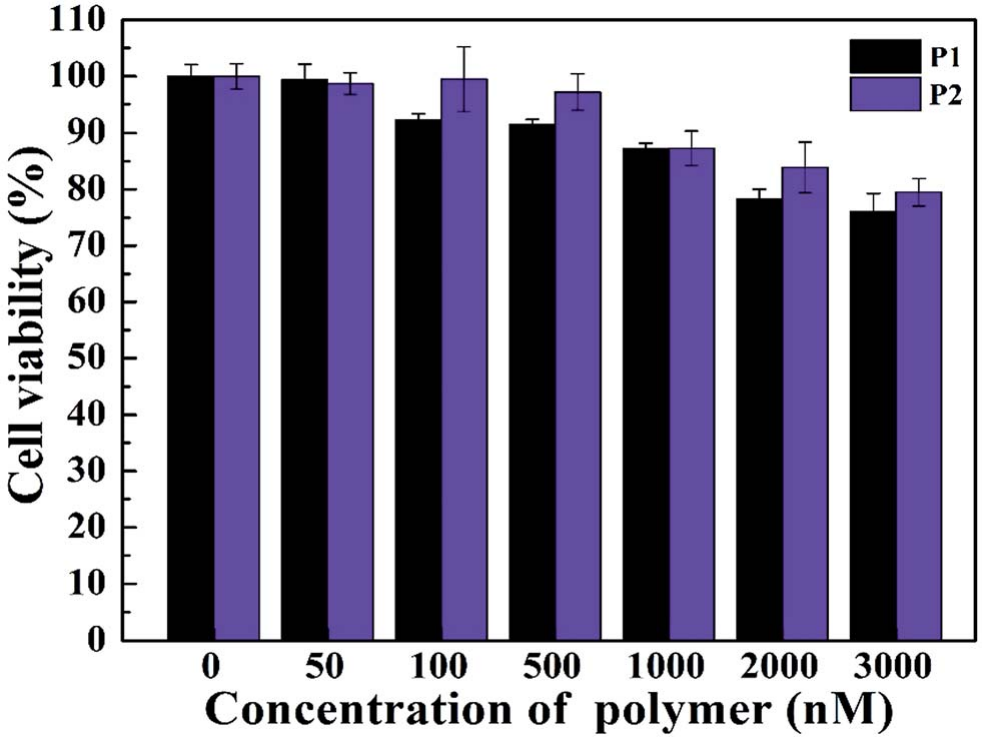
CCK-8 viability assay of HeLa cells treated with two different gene vectors under different concentrations.

The investigation on the cytotoxicity is important for gene transfection research. The cell viability of the vectors was evaluated by CCK-8 assay. When the concentration was up to 3 μM, the viability was still more than 75% (Fig. 4). The results showed that both P1 and P2 has low cytotoxicity and good biocompatibility. This experiment shows that vectors are biocompatible in terms of gene delivery, and our synthetic vectors achieve the desired effect to reduce the cytotoxicity.

**Fig. 5:**
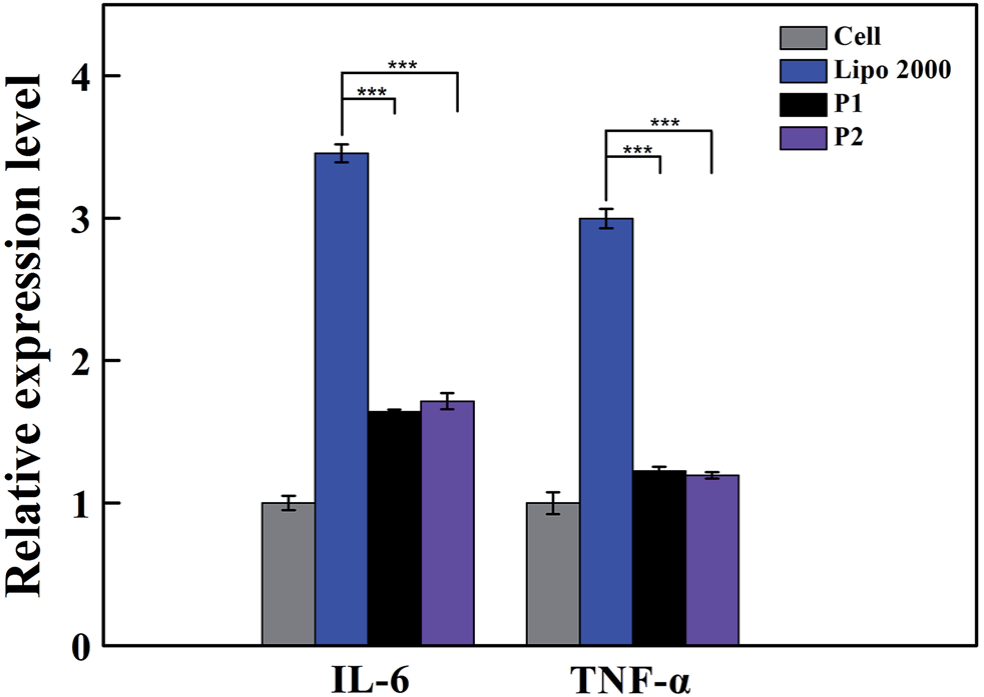
The relative expression levels of IL-6 and TNF-α in macrophage cells stimulated by P1 and P2 determined *via* qRT-PCR analysis. Cells without treatment were used as a negative control. The cells treated with lipofectamine 2000 were used as a positive control.

The biological effects of vectors released into immune cells were investigated to provide a theoretical basis for assessing the safety of vectors on living body immune system. The immunogenicity of vectors is also important to gene therapy application. The innate immune response can be stimulated by nanomaterials in macrophages *via* the production of inflammatory cytokines, including interleukin-1β (IL-1β), IL-6, IL-12, and tumor necrosis factor-α (TNF-α).^21,22^ qRT-PCR detection of cytokines expression in mRNA level is used to evaluate its immunogenicity.^23^ Our results illustrate the expression of IL-6 and TNF-α in RAW 264.7 stimulated by vectors. The cells treated with lipofectamine 2000 were used as positive control. Based on Fig. 5, all the two vectors exhibited less expression of IL-6 and TNF-α compared with lipofectamine 2000. For inflammatory cytokine IL-6, the immunogenicity of the vector was 1.6 times higher than that of the negative control group. The immunogenicity of the vector of the positive control group was 3.5 times of that in the experimental group. For the inflammatory cytokine TNF-α, the immunogenicity of the vector was 1.2 times of that in the negative control group, and the immunogenicity of the vector was 3.0 times of that in the positive control group. The results show that vectors hardly trigger the immune response to secrete cytokines, which confirms that the vector is of low immunogenicity and high biosecurity. However, when the macrophage incubated with polyplexes formed by the vectors and CpG DNA, which is known as high immunogenicity,^24^ the immune response cannot be covered by vectors (shown in Fig. S1 and S2†).

### 3.3 Evaluation of gene delivery

**Fig. 6:**
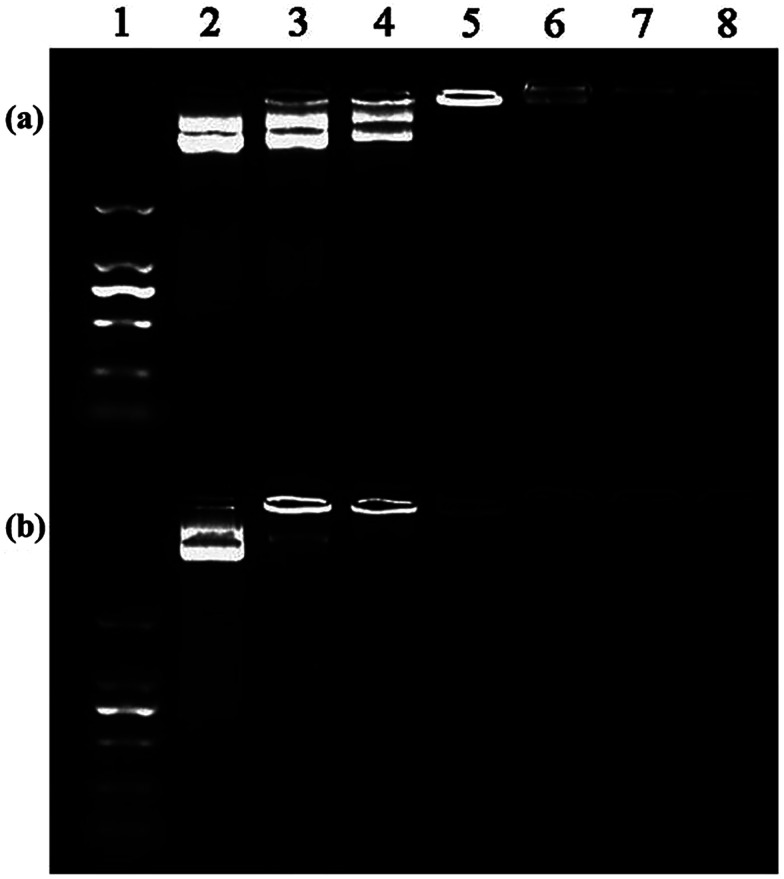
Agarose gel electrophoresis of P1/pDNA (a) and P2/pDNA (b) polyplexes at different N/P ratios. Lane 1: marker; lane 2: pDNA alone; lane 3: N/P = 0.25 : 1; lane 4: N/P = 0.5 : 1; lane 5: N/P = 1 : 1; lane 6: N/P = 2.5 : 1; lane 7: N/P = 5 : 1; and lane 8: N/P = 7 : 1.

**Fig. 7:**
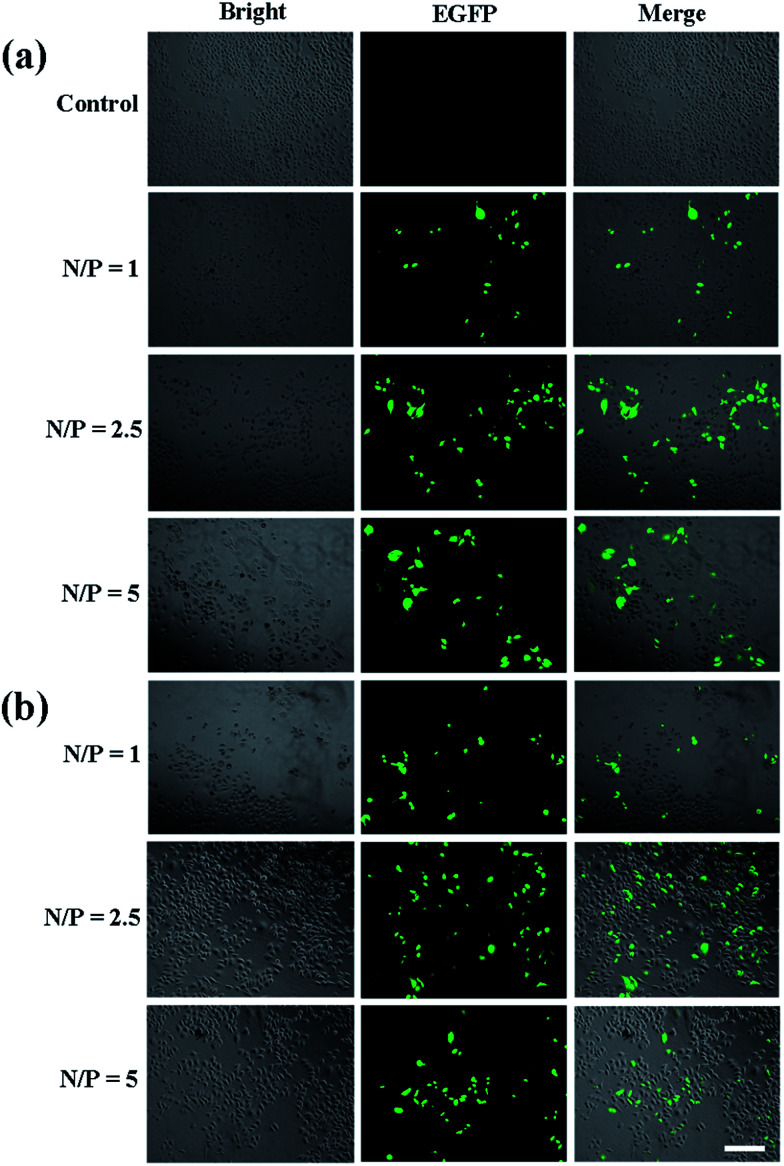
Fluorescence microscopic images of green fluorescent protein expression in HeLa cells using P1 (a) and P2 (b), respectively. The scale bar in each well represents 100 μm.

In order to systematically investigate the gene transfection capacity of the test vectors, we used flow cytometry and confocal microscopy to evaluate the efficiency quantitatively and qualitatively. Firstly, we need to identify the N/P ratios of vectors and nucleic acid. The PANOPA kit was used to determine the number of surface amine groups of the vectors for the calculation of N/P in the subsequent experiments. After mPEG modification, the number of terminal amine groups of P1 was 29.9, while the number of terminal amine groups of P2 was decreased to 21.6 after further connection with FA. Under different N/P conditions, the pDNA compaction ability of vectors can be confirmed by the gel retardation assay (Fig. 6). It is clear that the mobility of pDNA can be retarded by P2 at an N/P of 0.5, while P1 can retard the mobility at an N/P of 1. This indicates that P2 can effectively maintain the three dimensional spherical structure of dendrimer, improving the higher DNA compaction ability than P1. Considering that all the test vectors can completely retard the pDNA at an N/P ratio greater than 1 : 1, we chose the N/P of 1, 2.5 and 5 for the following experiments. The surface zeta potential and particle size are also measured, which can affect gene transfection and intracellular uptake experiments. The results are depicted in Table 1. It shows that the particle size of the polyplexes decreases continuously with the increase of N/P. The average particle size of the polyplexes is about 200 nm, which facilitates the polyplexes to transfect into cells. Besides, we can recognize that the zeta potential of the polyplexes formed by the two vectors and the pDNA are positive, indicating that vectors have positive charge as an integrity by completely compact the pDNA. Next, we investigated the *in vitro* transfection of the vector/pDNA polyplexes in HeLa cells by EGFP gene expression assay. Fluorescence microscopic images reveal that P2 vector allows more EGFP expression at all three N/P ratios, and the transfection efficiency at N/P ratio of 2.5 is the best (Fig. 7). This might be because the compaction ability of vectors at the N/P ratio of 2.5 is better than at the N/P ratio of 1, and the strong interaction between the vector and pDNA at an N/P ratio of 5 prevents pDNA to be released from the polyplexes. According to the Image J calculation, at N/P ratio of 2.5, the EGFP expression of the P2/pDNA polyplexes is 1.9 times higher than P1/pDNA polyplexes. It is notable that the amount of green fluorescent signal significantly increased after the surface modification of FA, which promotes the targeted delivery due to the interaction with the over-expressed FA receptors on the surface of HeLa cells. Our results imply that FA-mediated targeting mechanism can effectively improve the gene transfection capacity of the vector.

**Table 1:** Hydrodynamic size (a) and zeta potential (b) of P1, P2, P1/pDNA and P2/pDNA polyplexes at three different N/P ratios of 1 : 1, 2.5 : 1 and 5 : 1, respectively

Samples	NPs size ± SD (nm)	Zeta potential ± SD (mV)
P1	918.8 ± 33.1	29.6 ± 0.8
P1/pDNA (N/P = 1)	392.5 ± 42.5	11.8 ± 0.5
P1/pDNA (N/P = 2.5)	351.2 ± 44.2	14.5 ± 1.6
P1/pDNA (N/P = 5)	248.6 ± 20.3	17.3 ± 0.9
P2	966.7 ± 10.5	21.9 ± 0.6
P2/pDNA (N/P = 1)	344.5 ± 36.7	6.6 ± 0.2
P2/pDNA (N/P = 2.5)	186.5 ± 23.9	15.2 ± 0.2
P2/pDNA (N/P = 5)	144.9 ± 18.2	15.9 ± 0.4

**Fig. 8:**
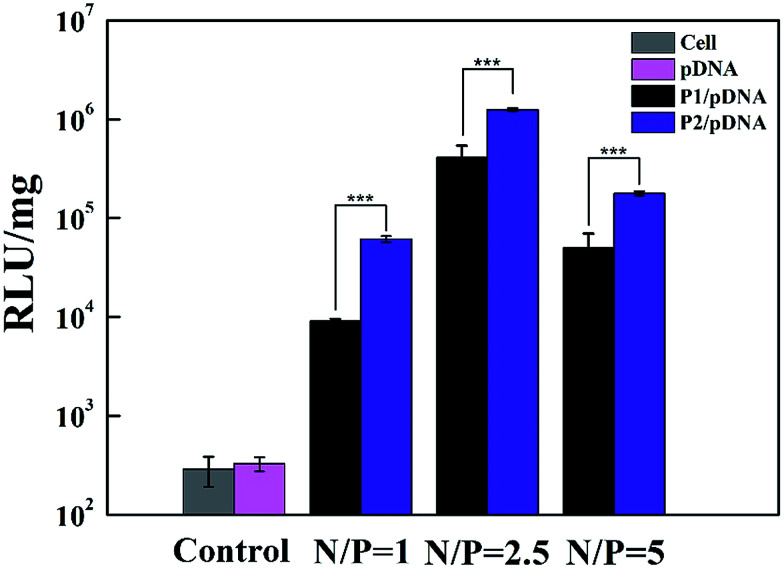
Luciferase reporter gene transfection efficiency of Au DENPs-mPEG/pDNA and Au DENPs-PEG-FA/pDNA polyplexes determined in HeLa cells at the N/P ratios of 1 : 1, 2.5 : 1 and 5 : 1, respectively. The cells treated with free pDNA and PBS-treated cells were used as control.

The gene transfection efficiency of the vectors was further explored quantitatively using Luc gene expression (Fig. 8). It appears that the transfection efficiency of P2 is higher than that of P1 in HeLa cells. At the N/P ratio of 2.5, the value (RLU per mg protein) of P2 is 3.7 times higher than that of P1, confirming that P2 vector is the most efficient carrier. This result is consistent with the EGFP gene expression. We believe that Au DENPs-PEG-FA can contribute to increased efficiency of gene transfection.

### 3.4 Cellular uptake of vector/pDNA polyplexes

**Fig. 9:**
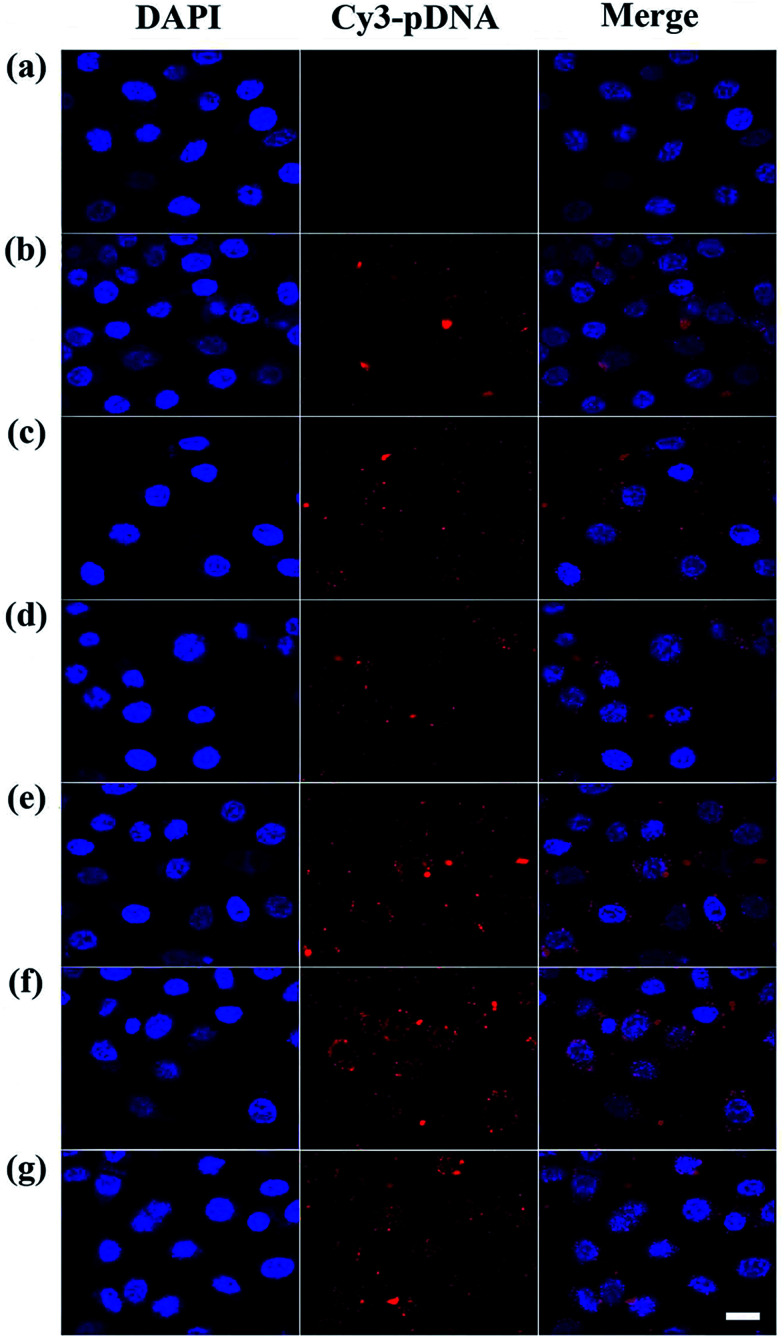
Laser confocal microscopy images (630×) of Cy3-pDNA with the Au DENPs-mPEG (b–d) and Au DENPs-PEG-FA (e–g) dendrimers delivered to HeLa cells at different N/P ratios of 1 : 1, 2.5 : 1 and 5 : 1 (blue: DAPI-stained cell nucleus; red: Cy3-labeled pDNA). Control (a) represents naked pDNA without vectors. The scale bar in each well represents 20 μm.

**Fig. 10:**
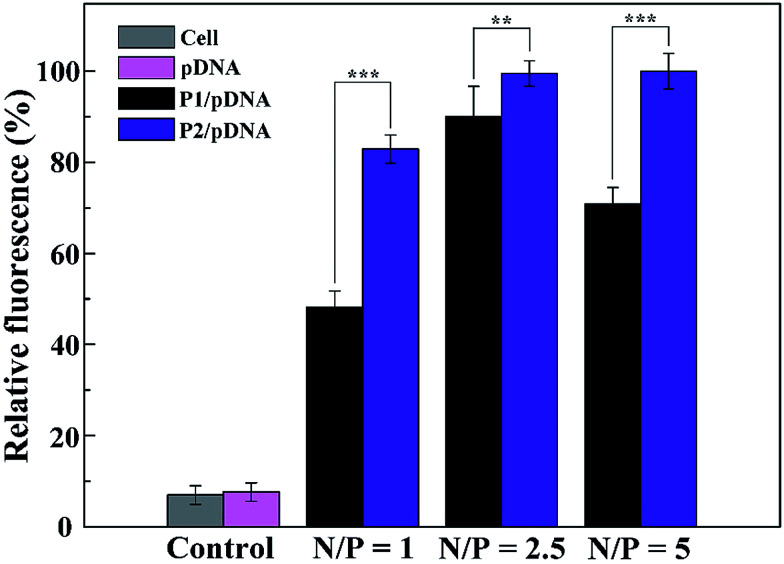
Flow cytometry measurement of the intracellular uptake of vectors/Cy3-labeled pDNA polyplexes at three different N/P ratios in HeLa cells. The cells without treatment and PBS-treated cells were used as controls.

To better understand the mechanism of gene delivery, we continued to investigate the cell uptake of vector/DNA polyplexes. As is known, a better cellular uptake capability of the vector/DNA polyplex is expected to result in enhanced gene delivery and expression. Confocal microscopy and flow cytometry were used to comprehensively evaluate cellular uptake behavior of the polyplexes using Cy3-labeled pDNA within the selected gate. As shown in Fig. 9, the red spots of Cy3 fluorescence in the cells are bright and numerous after incubation with the tested polyplexes, especially the P2 vector/DNA polyplex. Moreover, the Cy3-labeled pDNA distributed around the nucleus at N/P of 2.5 is large and numerous after the P2 compacted pDNA is transfected for 4 hours. The populations of the red fluorescent cells are up to 83%, 99% and 99% when P2 was used as a vector at the N/P ratio of 1, 2.5, 5, respectively (Fig. 10). The cellular uptake of P2/pDNA and is 1.73 times higher than that of P1/pDNA polyplex at the N/P ratio of 1. The results imply that the modification of FA is beneficial for enhanced cell uptake, which can support the above data of gene transfection. FA can more effectively compact pDNA into a small compound particles and surface of FA will possess appropriate charge to promote the interaction between the compounds and the cells, contributing to facilitate the targeted gene delivery.

**Fig. 11:**
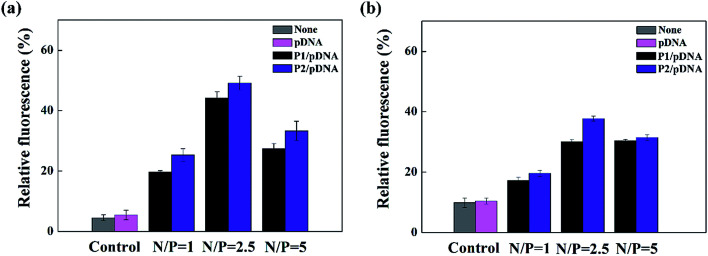
Flow cytometry measurement of the intracellular uptake of FAR blocked HeLa cells (a) and L929 cells (b) at different N/P ratios.

To further confirm the targeted-delivery effect of {(Au^0^)_50_-G5.NH_2_-PEG_10_-FA_5_}, L929 cells and FAR-blocked HeLa cells were used in gene transfection, which have less FAR on the cell surface.^16^ It is clear that the gene transfection efficiency of the polyplexes in these two kinds of cells was much lower than that in normal Hela cells (Fig. 11). What's more, there was no significant difference in cell uptake efficiency between P1 and P2. This indicates that the FA-targeted {(Au^0^)_50_-G5.NH_2_-PEG_10_-FA_5_} polyplexes enable targeted gene delivery to FAR-overexpressing cancer cells *via* receptor-mediated binding and intracellular uptake.

## 4 Conclusion

In summary, we synthesized a novel nonviral gene vector, Au DENPs-PEG-FA, to reduce the cytotoxicity of G5.NH_2_ and improve its gene transfection efficiency. Gel retardation assay demonstrate that Au DENPs-PEG-FA can effectively compact pDNA to form polyplexes. CCK-8 and qRT-PCR were used to evaluate the cytotoxicity and immunogenicity. The gene transfection efficiency of the vector was studied with two different pDNAs encoding Luc and EGFP respectively. The immunogenicity of vector is low and thus has biosecurity even when the gene transfection of vector is high. Our results clearly show that Au DENPs-PEG-FA are biocompatible and efficient for gene delivery. The modification of mPEG can increase the biocompatibility of the vector, and the targeting effect of FA enhances the transfection efficiency, which lays the foundation for further applications as a highly efficient gene carrier in biomedicine.

## Conflicts of interest

There are no conflicts to declare.

## Supplementary Material

RA-008-C7RA11901A-s001

## References

[cit1] Walther W., Stein U. (1999). Mol. Biotechnol..

[cit2] Winn S. R., Chen J. C., Gong X., Bartholomew S. V., Shreenivas S., Ozaki W. (2005). Orthod. Craniofac. Res..

[cit3] Li S. D., Huang L. (2007). J. Controlled Release.

[cit4] Behr J. P. (1994). Bioconjugate Chem..

[cit5] Qi R., Gao Y., Tang Y., He R. R., Liu T. L., He Y., Sun S., Li B. Y., Li Y. B., Liu G. (2009). AAPS J..

[cit6] Majoros I. J., Keszler B., Woehler S., Bull T., Baker J. R. (2003). Macromolecules.

[cit7] Tanaka N., Fukutome T., Hosoya K., Kimata K., Araki T. (1995). J. Chromatogr. A.

[cit8] Jevprasesphant R., Penny J., Attwood D., McKeown N. B., D'Emanuele A. (2003). Pharm. Res..

[cit9] Luo D., Haverstick K., Belcheva N., Han E., Saltzman W. M. (2002). Macromolecules.

[cit10] Marques J. T., Williams B. R. G. (2005). Nat. Biotechnol..

[cit11] Knuschke T., Bayer W., Rotan O., Sokolova V., Wadwa M., Kirschning C. J., Hansen W., Dittmer U., Epple M., Buer J., Westendorf A. M. (2014). Nanomedicine.

[cit12] Cao X. Y., Lu X. G., Wang D. L., Jia F., Tan X. Y., Corley M., Chen X. Y., Zhang K. (2017). Small.

[cit13] Zhang Q., Kusaka Y., Sato K., Nakakuki K., Kohyama N., Donaldson K. (1998). J. Toxicol. Environ. Health, Part A.

[cit14] Ding L., Stilwell J., Zhang T., Elboudwarej O., Jiang H., Selegue J. P., Cooke P. A., Gray J. W., Chen F. F. (2005). Nano Lett..

[cit15] Shvedova A. A., Kisin E. R., Mercer R., Murray A. R., Johnson V. J., Potapovich A. I., Tyurina Y. Y., Gorelik O., Arepalli S., Berry D. S., Hubbs A. F., Antonini J., Evans D. E., Ku B. K., Ramsey D., Maynard A., Kagan V. E., Castranova V., Baron P. (2005). Am. J. Physiol.: Lung Cell. Mol. Physiol..

[cit16] Xiao T. Y., Hou W. X., Cao X. Y., Wen S. H., Shen M. W., Shi X. Y. (2013). Biomater. Sci..

[cit17] Shi X. Y., Wang S. H., Inhan L., Shen M. W., Baker Jr J. R. (2009). Biopolymers.

[cit18] Schmittgen T., Zakrajsek B. (2001). J. Biochem. Biophys. Methods.

[cit19] Shan Y. B., Luo T., Peng C., Sheng R. L., Cao A. M., Cao X. Y., Shen M. W., Guo R., Tomas H., Shi X. Y. (2011). Biomaterials.

[cit20] Kong L., Alves C. S., Hou W., Qiu J., Möhwald H., Tomas H., Shi X. Y. (2015). ACS Appl. Mater. Interfaces.

[cit21] Alfarsi M. A., Hamlet S. M., Ivanovski S. (2014). J. Biomed. Mater. Res., Part A.

[cit22] Szlosarek P., Charles K. A., Balkwill F. R. (2006). Eur. J. Cancer.

[cit23] Hajam I. A., Dar P. A., Appavoo E., Kishore S., Bhanuprakash V., Ganesh K. (2015). PLoS One.

[cit24] Sandler A. D., Chihara H., Kobayashi G., Zhu X., Miller M. A., Scott D. L., Krieg A. M. (2003). Cancer Res..

