# Supplementary material for: PEGylated dendrimer-entrapped gold nanoparticles with low immunogenicity for targeted gene delivery
Source: RSC Adv. 2018 Jan 3;8(3):1265–73. doi: 10.1039/c7ra11901a (PMC9076948; doi:10.1039/c7ra11901a)

Electronic Supplementary Material (ESI) for RSC Advances.
This journal is © The Royal Society of Chemistry 2017

Supporting information

PEGylated dendrimer-entrapped gold nanoparticles with low immunogenicity for targeted gene delivery

Bei Xu, Aijun Li, Xinxin Hao, Rui Guo, Xiangyang Shi* and Xueyan Cao*

College of Chemistry, Chemical Engineering and Biotechnology, Donghua University, Shanghai 201620, People's Republic of China

Figure S1. The relative expression levels of IL-6 and TNF-*α* in macrophage cells stimulated by vectors/pDNA complexes determined by qRT-PCR analysis. Cells treated with pDNA was used as a negative control (Cell). The cells treated with lipofectamine 2000 and pDNA was used as a positive control (Lipo/pDNA).

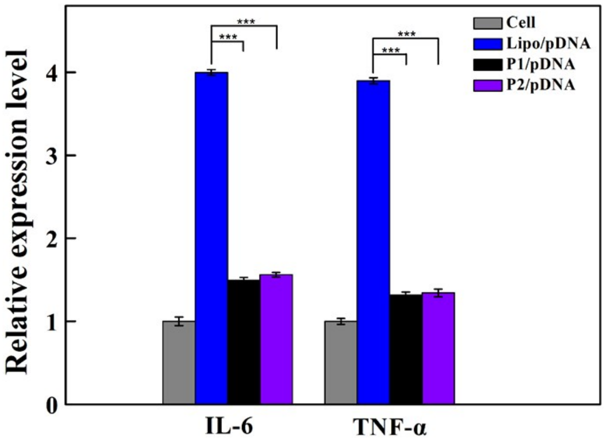

Figure S2. The relative expression levels of IL-6 and TNF-*α* in macrophage cells stimulated by vectors/CpG DNA complexes determined by qRT-PCR analysis. Cells without treatment was used as a negative control (Cell). The cells treated with lipofectamine 2000 and CpG DNA was used as a positive control (Lipo/CpG).

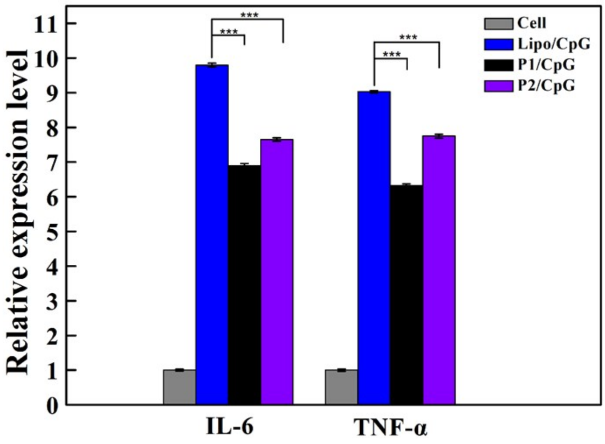

Supplement: RA-008-C7RA11901A-s001 [file RA-008-C7RA11901A-s001.pdf]
